# Advances in the use of ECMO in oncology patient

**DOI:** 10.1002/cam4.6288

**Published:** 2023-07-17

**Authors:** Xiangnan Teng, Jiali Wu, Jing Liao, Shanling Xu

**Affiliations:** ^1^ Department of Critical Care Medicine, Sichuan Cancer Hospital & Institute, Sichuan Cancer Center, School of Medicine University of Electronic Science and Technology of China Chengdu China; ^2^ Department of Respiratory and Critical Care Medicine The Affiliated Hospital of Southwest Medical University Luzhou China

**Keywords:** cancer patient, extracorporeal membrane oxygenation, intensive care unit

## Abstract

**Background:**

Over the past decade, ECMO has provided temporary cardiopulmonary support to an increasing number of patients, but the use of extracorporeal membrane oxygenation (ECMO) to provide temporary respiratory and circulatory support to adult patients with malignancy remains controversial.

**Objectives:**

This paper reviews the specific use of extracorporeal membrane oxygenation (ECMO) in oncology patients.

**Methods:**

We searched PubMed, CINAHL, Cochrane Library, and Embase databases for studies on the use of ECMO in cancer patients between 1998 and 2022. Twenty‐four retrospective, prospective, and case reports were included. The primary outcome was survival during extracorporeal membrane oxygenation.

**Results:**

Most studies suggest that ECMO can be used in oncology patients requiring life support during surgery, solid tumor patients with respiratory failure, and hematological tumor patients requiring ECOM as a supportive means of chemotherapy; however, in patients with hematologic oncology undergoing hematopoietic stem cell transplantation, there was no clear benefit after the use of ECMO.

**Conclusion:**

Current research suggests that ECMO may be considered as a salvage support in specific cancer patients. Future studies should include larger sample sizes than those already conducted, including studies on efficacy, adverse events, and health.

## 1 INTRODUCTION

With the continuous development and advancement of medical technology and drugs, the mortality rate of solid malignancies and hematological malignancies patients is gradually decreasing.^1^ Improved efficacy and supportive measures have also changed our attitudes towards patients entering the intensive care unit (ICU). In the meantime, a consensus document highlights the need for full‐code ICU management (without limitations of ICU resources) for all critically ill cancer patients if the prognosis of the underlying malignancy is long‐term survival.^2^ Some patients who survive ICU support have a high quality of life after being discharged from the hospital and can continue their anticancer treatment; their long‐term survival depends on the status of the underlying disease and is indistinguishable from oncology patients not admitted to the ICU.^3^, ^4^ Nevertheless, mortality remains high in critically ill oncology patients and is associated with complications, such as infection, treatment‐related reactions, and disease progression.^5^, ^6^ Extracorporeal membrane oxygenation (ECMO) is also used in severe cases of respiratory distress syndrome such as those caused by neocoronavirus and influenza A (H1N1).^5^, ^6^, ^7^

ECMO was developed outside of the operating room as an extension of the use of cardiopulmonary bypass (CPB) and was initially used for surgical patients who did not tolerate separation from CPB. CPB provides support to patients undergoing surgery for a few minutes to several hours. In addition to cardiac surgery, it is also used in major vascular surgery, neurosurgery, and transplantation. The use of ECMO in the treatment of patients with respiratory, cardiac, or combined failure can last several days or weeks. The use of ECMO as a potentially lifesaving intervention is becoming increasingly popular and its applications are becoming more diverse. It was initially used in the 1950s to provide support to patients undergoing cardiopulmonary bypass surgery.^8^ Depending on the indication, there are two main configurations of ECMO: VV in patients with severe respiratory failure intravenous ECMO (VV) ECMO, and ECMO (VA) for severe heart failure. Venovenous ECMO (VV‐ECMO) only provides respiratory support to patients. It is divided into a single cannula approach and a two‐cannulation approach, with a single cannula approach flowing through the vena cava or right atrium outflow through circulation and then into the right atrium. The two‐cannulation approaches flow from the common femoral vein and through the right internal jugular or femoral vein.^9^ Venoarterial ECMO (VA‐ECMO) provides both respiratory and hemodynamic support. The VA‐ECMO circuit is connected parallel to the heart and lungs, whereas the VV‐ECMO circuit is connected in series with the heart and lungs.^10^ The Peripheral VA‐ECMO cannulation approach is used when blood is drawn from the right atrium or vena cava and returned to the femoral artery, axillary, or carotid artery in the arterial system.^11^ As technology grows, its use has expanded. In particular, ECMO for the support of cases of severe respiratory distress syndrome has caused a dramatic rise in the use of ECMO for life support in oncology patients in the last decade.^5^, ^6^, ^7^

However, the use of ECMO in patients with malignant tumors remains controversial. First, immunosuppression is listed as a relative risk for ECMO by the Extracorporeal Life Support Organization (ELSO), which manages the ECMO record of operational ELSO centers globally.^12^ Most oncology patients are considered immunosuppressed; many suffer from thrombocytopenia or plasma coagulation disorders, and many are receiving chemotherapy or immunomodulatory therapies, all of which may interfere with extracorporeal circulation and are thus considered contraindications for ECMO.^13^ Secondly, cancer progression cannot be interfered with or reversed. We can prolong the life cycle of patients with some treatments; however, we have not yet achieved a cure. Finally, the arbitrary use of these resources may place some financial strain on the health care system.^10^, ^14^ However, this view has been challenged by retrospective studies showing that ECMO provides an alternative treatment for patients with solid tumors that are unsuitable for conventional surgical resection, thereby improving survival in this group of patients.^15^ Furthermore, the most common reason that patients with malignant hematological tumors (such as leukemia, lymphoma, and multiple myeloma) need to be admitted to the ICU is acute respiratory failure.^16^ Although patients with hematological tumors have benefited from chemotherapy and hematopoietic stem cell transplantation in recent years, more than 50% of hospitalized patients still require mechanical ventilation.^17^, ^18^ In such cases, ECMO may be considered^19^; ECMO can help patients with tumor lysis syndrome by stabilizing their condition alongside chemotherapy.^20^

Therefore, the purpose of this review was to describe how ECMO is used to support different types of oncology patients, evaluate the prognosis of oncology patients who receive ECMO, and identify the types of oncology patients who could benefit from ECMO.

## 2 METHODS

We conducted a comprehensive literature search of PubMed, CINAHL, Cochrane Library, and Embase databases from 1998 to 2022. Databases were electronically searched for relevant publications using combinations of the following medical subject headings (MeSH) and keywords (“Venoarterial Extracorporeal Membrane Oxygenation” or “Venovenous Extracorporeal Membrane Oxygenation”) and (“Tumor” or “Cancer” or “Malignancies”). In addition, a hand search of the references in included full texts was performed to identify additional studies for inclusion.

**FIGURE 1:**
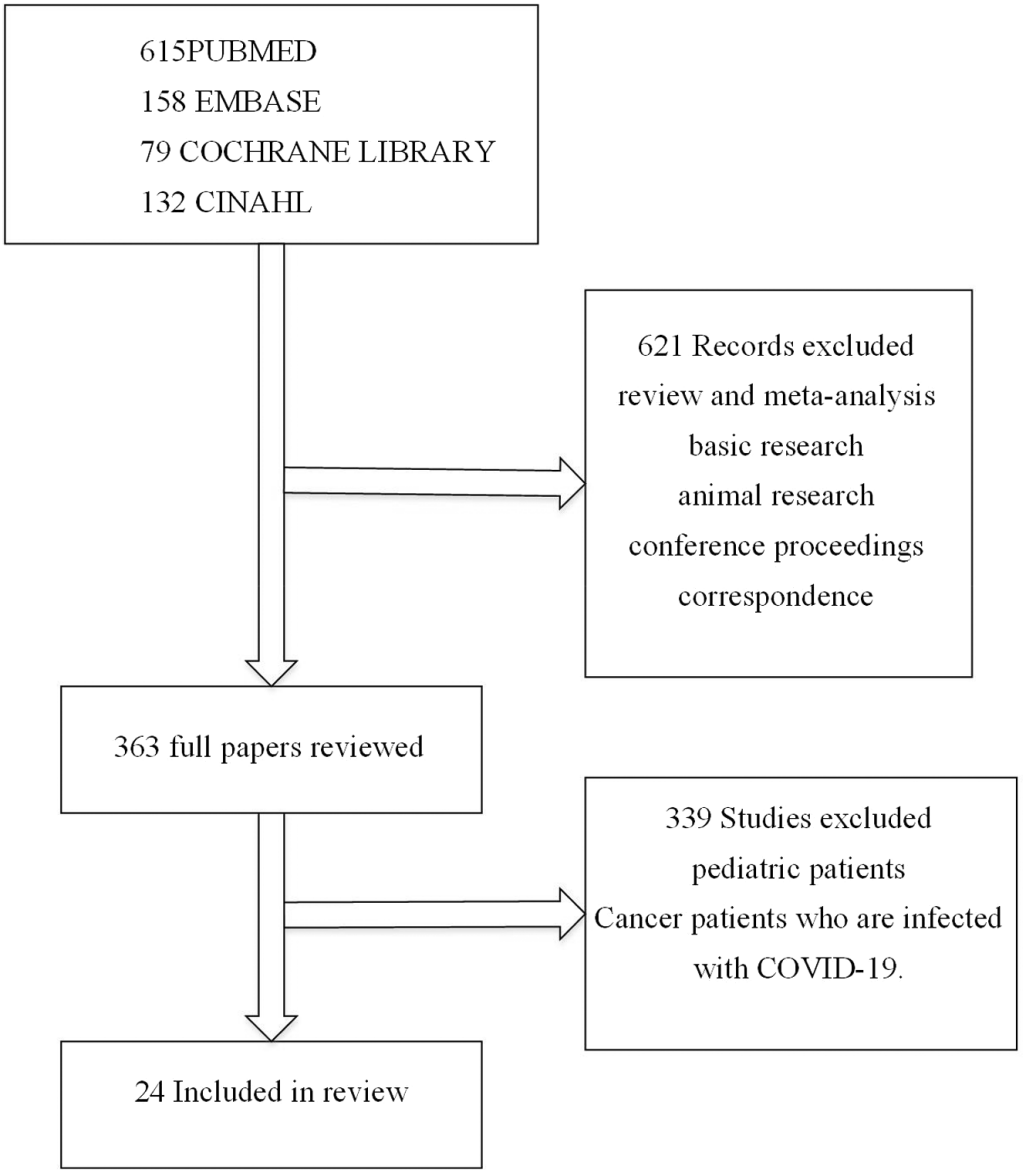
Flowchart of study selection.

We initially identified 984 publications in the extracorporeal membrane oxygenation and cancer search. All studies were screened for eligibility by reading the title and abstract, and a total of 621 publications were excluded due to the types including review and meta‐analysis, basic research, animal research, conference proceedings, and correspondence, leaving 363 publications for evaluation. Three hundred thirty‐nine studies were excluded based on the evaluation of both the abstract and full text, as they involved pediatric oncology patients and adult oncology patients who had concurrent COVID‐19 infection. At last, we performed a full‐text evaluation of 24 papers (Figure 1). Twenty‐four studies of ECMO support for oncology patients were included in this review. Ten of the included studies are retrospective, one is prospective, and thirteen are case reports. Further information on these studies is provided in Table 1.

**TABLE 1:** Publications on extracorporeal membrane oxygenation and cancer included in the review.

Author	Characteristics of studies	Study objective	Study outcome
Gow et al.^21^	Retrospective study	Consider whether extracorporeal life support can be used for patients with malignancy after conventional treatment has failed	A total of 72 patients with tumors were included: 47 with solid tumors, 21 with hematological tumors, and 4 with hematopoietic stem cell transplants. 32% of the patients survived to discharge, and 61% died from ELCS
Wu et al.^22^	Retrospective study	Is VV ECLS a salvageable support for oncology patients in acute respiratory failure	Fourteen patients with tumors were included: 13 with solid tumors and one with hematological tumors. Four patients survived and were discharged
Kochanek et al.^23^	Retrospective study	The aim is to investigate whether VV‐ECMO is beneficial in supporting patients with cancer and severe respiratory failure	With an overall survival rate of 26.8% at 60 days, the value of ECMO in patients with cancer has not yet been determined, and further research is needed
Wohlfarth et al.^24^	Retrospective study	Report the outcome of patients with hematological oncology suffering from acute respiratory failure receiving ECMO support in the intensive care unit	A total of 14 patients were included in the study. Out of these, 7 patients survived. The median follow‐up time for these 7 patients was 36 months
Kang et al.^25^	Retrospective study	Exploring the clinical outcomes of patients with hematologic malignancies who have been treated with ECMO	Patients with hematologic malignancies who underwent ECMO had lower survival rates compared with immunocompetent patients. However, there were no significant differences in the complications experienced by the two groups
Liao et al.^26^	Case report	Report on supporting patients with idiopathic pneumonia syndrome after allogeneic hematopoietic stem cell transplantation through intravenous ECMO	Patient successfully supported by intravenous ECMO
Koinuma et al.^27^	Case report	Use of ECOM to maintain lung ventilation in patients with hematopoietic stem cell‐transplanted blood systems to treat Idiopathic pneumonia syndrome	The patient was in remission after treatment with ECOM support but died soon after extubation
Wohlfarth et al.^28^	Retrospective study	Investigating the use of ECMO as a means of providing resuscitation support for recipients of ex vivo hematopoietic stem cell transplants	The survival rate for patients who started receiving extracorporeal membrane oxygenation within 240 days of allogeneic HSCT was 46%
Stecher et al.^29^	Retrospective study	The aim is to evaluate the viability of using ECMO as a treatment for cancer patients who have significant leukocytopenia and thrombocytopenia	20% of the patients survived until hospital discharge
Spaggiari et al.^30^	Prospective study	To evaluate whether ECMO can be used as a method of lung ventilation during tracheal in Tracheal sleeve pneumonectomy	All the patients survived and did not have any postoperative complications
Schiff et al.^31^	Case report	Report the use of ECMO as a perioperative support method for a patient undergoing two surgical procedures	The patient was being weaned off ECMO on the third day following the operation and was discharged from the hospital on the 15th day after the operation
Rinieri et al.^32^	Retrospective study	Evaluate the benefits and complications of using ECMO as respiratory support during major trachea‐bronchial surgery and single‐lung procedures	The mortality rate within 30 days for patients who received ECMO as complete replacement for extracorporeal circulation during thoracic surgery was 67%
Redwan et al.^33^	Retrospective study	Report on the specific use of extracorporeal membrane oxygenation in thoracic surgery	All six patients successfully underwent the extracorporeal life support procedure and avoided other complications that may have arisen from extracorporeal circulation, such as CPB
Sauneuf et al.^34^	Retrospective study	Describe the outcomes of patients with pheochromocytoma who were admitted to the ICU with and without the use of ECMO	The crude mortality rate among patients who received extracorporeal membrane oxygenation treatment was 22% within 90 days. The findings indicated that there was no statistically significant difference in mortality between patients who received ECMO treatment and those who did not
Novellis et al.^35^	Case report	The patient with stage cIIA lung cancer, arterial infiltration, and severe postischemic dilated cardiomyopathy (ejection fraction of 0.23) underwent left upper lobectomy and pulmonary angioplasty with the use of VA‐ECMO as cardiopulmonary support	The procedure was completed successfully with the hemodynamic conditions provided by ECMO, and the patient was discharged on the third day after the operation
Meltzer et al.^36^	Case report	Report on the use of VA‐ECMO to provide cardiopulmonary support for chemotherapy in patients with lymphoma	The patient achieved complete radiographic remission after receiving chemotherapy with ECMO support but unfortunately passed away due to infection 1 year later
Allain et al.^37^	Case report	A report on chemotherapy with ECMO support in patients with primary cardiac lymphomas(PCLs) following the onset of cardiogenic shock	The patient had successful chemotherapy and recovered
Chung et al.^38^	Case report	Report on a patient with metastatic choriocarcinoma who was admitted to the intensive care unit due to hemodynamic instability. The patient received chemotherapy after VA‐ECMO	The patient underwent conventional chemotherapy treatment with ECMO support and responded positively to the treatment. She was successfully discharged from the hospital after completing the chemotherapy
Rotz et al.^39^	Case report	Report of a young man with primary mediastinal B‐cell lymphoma with the invasion of the trachea, who completed chemotherapy during ECMO support	The patient underwent conventional chemotherapy treatment with ECMO support and responded positively to the treatment. They were successfully discharged from the hospital after completing the chemotherapy
Pendleton et al.^40^	Case report	A patient with esophageal adenoid cystic carcinoma underwent surgical resection while receiving intravenous extracorporeal membrane oxygenation due to the tumor's invasion of the trachea	With the support of ECMO, the patient underwent resection of the primary tumor, which had also invaded the trachea
Huang et al.^41^	Case report	A young woman with a mediastinal hemangioma had refractory hemoptysis due to the tumor. After multiple bronchial artery embolizations failed, she was treated surgically with the support of VV‐ECMO	With the support of ECMO, the patient completed mediastinal hemangioma resection and right upper lobe sleeve resection was performed
Smith et al.^42^	Case report	Report on two patients who underwent surgical resection for obstructive papilloma with ECMO support	Both patients underwent tumor resection with the support of ECMO; however, the first patient experienced post‐ECMO complications
Takeda et al.^43^	Case report	A 19‐year‐old male patient had a large anterior mediastinal tumor. During the operation, ECMO oxygen support was used because of severe hypoxemia	The patient underwent resection with respiratory support from ECMO
Stewart et al.^44^	Case report	A patient with a large mass in the mediastinum was described, and the mass was causing compression of the airway. The patient received treatment with antineoplastic therapy and was supported with cardiopulmonary assistance on ECMO	The patient was successfully discharged after receiving antitumor treatment with cardiopulmonary support on ECMO, and the tumor shrank

### 2.1 Respiratory support for patients with malignant tumors

With the growing capacity to inhibit tumor growth, new cancer treatment options have greatly slowed the progression and recurrence of cancer, and promoted remission. However, as life expectancy increases, the risk of acute respiratory failure in cancer patients also increases. Solid tumors and hematological malignancies differ in their pathogenesis, treatment, and patient prognosis. Therefore, it is necessary to discuss these two groups of patients separately.

#### 2.1.1 Respiratory support for patients with solid malignancies

In 2010, Gow et al. retrospectively analyzed 72 adult patients with malignancies, 47 of whom had solid tumors. Among these patients, the extubation rate was 35% (17) and the discharge rate was 29% (7).^45^ When severely ill cancer patients require invasive ventilator assistance, their prognosis is dismal (20%–30% survival rate).^46^, ^47^ However, since 2015, Wu et al. suggest that ECMO should be offered to patients with cancer who are suffering from respiratory failure. Thirteen oncology patients who received ECMO after anticancer treatment were included in this retrospective study. The median follow‐up was 11 months, and only four patients survived during this period (partial remission), two of whom died of recurrent cancer. Patients with recurrent or progressive malignancies who have severe granulocytopenia cancer did not benefit from ECMO treatment for acute respiratory failure, according to the authors.^48^ Kochanek et al., in 2022, mentioned that cancers associated with severe respiratory failure had a poor prognosis after ECMO treatment. Patients with progressive disease are not recommended to undergo ECMO, and the survival rate for solid body tumors is 30% at 60 days^49^ (Table 2). Compared with the study by Wu et al, relevant physiological parameters (e.g., neutrophil and platelet counts) improved in oncology patients after ECMO use, although patient survival was lower in the study by Gow et al. However, whether this change in parameters is related to patient prognosis requires further investigation. Since 2015, discharge rates for oncology patients have increased in several studies compared with previous studies, which may be related to the increasing number of treatment options for oncology patients and advances in ECMO technology. Meanwhile, researchers have suggested supportive care for solid tumor patients with high functional status (functional status score (PS) <2 or good long‐term prognosis).

**TABLE 2:** Clinical features of patients with solid tumors.

Reference	Type of tumor	Patients included	Period	ECMO weaning	Survival on POD 90	Reason for death	Complications
Stewart et al.^44^	Solid tumor	1	1998	1	1	–	No
Takeda et al.^43^	Solid tumor	1	1999	1	1	–	No
Lang et al.^60^	Solid tumor	9	2011	8	8	Brain metastasis Hepatic and small bowel necrosis	Hepatic and small bowel necrosis
Huang et al.^41^	Solid tumor	1	2021	1	1	–	No
Schiff et al.^31^	Solid tumor	1	2013	1	1	–	No
Spaggiari et al.^15^	Solid tumor	51	2021	51	51	–	Hemothorax
Gow et al.^45^	Solid tumor	47	2010	30	33	–	Infarction
Wu et al.^48^	Solid tumor	13	2015	7	5	–	Hemothorax
Kochanek et al.^49^	Solid tumor	159	2022	31	47	–	Hemothorax
Sauneuf et al.^34^	Solid tumor	34	2017	8	9	–	Hemothorax
Audrey et al.^62^	Solid tumor	1	2022	–	1	–	No
Weizhao et al.^56^	Solid tumor	1	2011	1	1	–	No
Simth et al.^42^	Solid tumor	2	2009	2	–	–	Neuropraxia of the lateral cutaneous nerve of the right thigh.

#### 2.1.2 Respiratory support for patients with hematological malignancies

Poor prognosis for patients with hematological malignancies who present with severe respiratory failure.^50^

The results of ECMO support in patients with hematological malignancies are inconsistent. Among 72 adult patients with malignancies studied by Gow et al., 21 had hematological malignancies, and four underwent HSCT. Accordingly, 32% of patients with hematological tumors survived hospital discharge, supporting the general view that ECMO is not indicated for these patients.^45^ However, Wohlfarth et al. provided a different view, arguing that ECMO can be used for specific patients with hematologic malignancies in respiratory failure, and is associated with long‐term disease‐free survival. During a 36‐month follow‐up period, 7 patients survived, including one in complete remission, one in partial remission, and one in relapse.^16^ However, since 2015, there has been enough evidence of ECMO improving the prognosis of patients with hematological malignancies. Kang et al. reviewed the data of patients who received ECMO, 15 of whom had hematologic malignancies and 33 were immunocompetent. The prognosis was poorer for ECMO patients with hematological malignancies than for those who were immunocompetent; however, there was no significant difference in the complications that occurred between the two groups. This study suggests that patients who require emergency support require further consideration^51^ (Table 3). One may consider why the study by Wohlfarth et al. showed a higher long‐term survival rate than that by Gow et al. The reasons for this could be related to biases in patient selection, disease status, and differences in hospital‐based treatment levels. Due to treatment differences and subgroups of blood diseases between the two study populations, it is difficult to simply compare the results of these studies. For example, among the patients studied by Gow et al., the majority had acute leukemia, whereas the majority in Wohlfarth et al. had lymphoma. In comparison to other hematological malignancies, acute leukemia has a poor prognosis.

**TABLE 3:** Clinical features of patients with hematologic malignancies.

Reference	Type of tumor	Patients included	period	ECMO weaning	Survival on POD 90	Cause of death	Complications
Gow et al.^21^	Hematologic malignancy	22	2010	9			
Wohlfarth et al.^24^	Hematologic malignancy	14	2014	7	7		Hemothorax
Kang et al.^25^	Hematologic malignancy	15	2015	0	0		Infection
Liao et al.^26^	Hematologic malignancy	1	2013	1	1		No
Koinuma et al.^27^	Hematologic malignancy	1	2014	0	0		Hemothorax and Infection
Wohlfarth et al.^28^	Hematologic malignancy	37	2017	15	7		Hemothorax
Stecher et al.^29^	Hematologic malignancy	25	2018	5	1		ARDS
Meltzer et al.^36^	Hematologic malignancy	1	2014	1	1		No
Allain et al.^37^	Hematologic malignancy	1	2015	1	1		No
Chung et al.^38^	Hematologic malignancy	1	2016	1	1		No
Rotz et al.^39^	Hematologic malignancy	1	2020	1	1		No

#### 2.1.3 Respiratory support for patients after hematopoietic stem cell transplantation

A variety of malignant and nonmalignant hematologic conditions are being increasingly treated with allogeneic hematopoietic stem cell transplantation (ASCT). In 2014, there were 8000 ASCTs performed in the United States and 16,000 in Europe.^52^, ^53^ However, as many as 16% of patients develop acute respiratory distress syndrome (ARDS) in the first year following ASCT, and three‐quarters develop severe ARDS.^54^, ^55^ This finding suggests that ECMO should be considered in ASCT recipients with severe ARDS.

In 2013, a B‐cell acute leukocyte patient treated with hematopoietic stem cell transplant underwent ECMO support and showed a complete absence of respiratory failure and other symptoms. It is clear from this case that ECMO may be successful in treating such patients, although its prognosis has not yet been addressed.^26^ However, this was not the case in the 2014 study by Koinuma et al., in which a patient with leukemia who had received hematopoietic stem cell transplantation and ECMO respiratory support for 11 days died shortly after extubation.^56^ According to a retrospective study conducted in 2017, 19% (*n* = 7) of patients received ECMO for respiratory failure after ASCT. Only one patient who started ECMO within 240 days died, whereas six patients who started ECMO after 240 days survived.^57^ A retrospective study in 2018 analyzed 25 patients, 11 of whom had undergone HSCT. Overall, 68% of these patients died, 20% (5) survived until discharge, and all patients who underwent HSCT were died. Therefore, the use of ECMO to support ARDS in patients receiving ASCT needs to be considered with caution.^29^ In the retrospective studies described above, it appears that except in a few cases, patients treated with ECMO support with ASCT complicated by respiratory failure had a higher mortality rate. However, in a 2017 study, the authors mentioned that 240 days after ASCT, patients had a better prognosis with ECMO than those within 240 days, which was related to the low immune status of patients who had just finished ASCT treatment.

### 2.2 Adjunctive perioperative support for patients with solid tumors

Extracorporeal lung support (ECLS) is gaining acceptance for thoracic surgery. It has been increasingly used in oncological thoracic surgery since its initial use in lung transplantations. Classification can be based on the patient's treatment needs and can be divided into surgery without a history of extensive resection on the contralateral side or patients with severely impaired lung function and procedures requiring both ventilatory and hemodynamic instability support. ECMO has been used as a means of support to assist patients with breathing since 1998 when it successfully supported adult men with severe acute mediastinal tumors causing airway compressions.^44^, ^58^ In 2021, Huang et al. similarly described a case in which a patient underwent mediastinal tumor resection with ECMO support.^59^ Lang et al. retrospectively analyzed nine patients with thoracic malignancies between 2001 and 2011. All nine patients underwent complex tracheobronchial resection with ECMO support, and eight patients (89%) achieved R0 resection.^60^ In 2009, Smith et al. similarly described two patients who underwent successful resection of bronchial tumors with ECMO support.^42^

Spaggiari et al. retrospectively analyzed six cases in which ECMO was assisted by tracheal sleeve pneumonectomy (TPS) to improve ventilation, and patients with lung cancer received complete surgical treatment. The follow‐up period was 8.4 months and all patients survived.^15^ The use of ECMO is not only limited to bronchial or lung malignancies but can also be used to help patients with esophageal cancer. This is based on an isolated case but may prove significant. Approximately 12 years ago, the patient underwent left pneumonectomy, followed by chemotherapy and radiotherapy for non‐small‐cell lung cancer. Although lung cancer was prominent, esophageal cancer could not be ruled out. According to the oncologist, surgical resection is recommended for radical esophageal cancer. In the case of a patient who had only one lung, and with the postoperative complications of esophageal cancer already high, the use of ECMO to support the surgery was proposed. Patients with multiple or more complex comorbidities may benefit from ECMO during major thoracic surgery.^61^ Similarly, Audrey et al. described a patient with esophageal adenoid cystic carcinoma that invaded the trachea. The patient underwent tumor resection and tracheal repair while on ECMO.^62^ Pheochromocytoma crises are rare, but with sudden onset and nonspecific symptoms, nearly a quarter of the patients die. Salvage ECMO is used in most severe cases, effectively helping patients overcome circulatory failure. According to a French study by Rinieri et al.'s from 2015, 36 patients were divided into three separate groups. The 30‐day mortality rate in the group receiving full ECMO support was only 7%, which was statistically significant compared with the other two groups. ECMO can also be used to support conventional lung ventilation, according to the authors.^63^ Six patients underwent pneumonectomy using both high‐ and low‐flow modes. Redwan et al. reported that VV‐ECMO could provide lung support in different settings.^64^ In 2017, a French multicenter cohort study included 34 patients with pheochromocytoma (the largest series of patients with pheochromocytoma), of which 41% (14) used ECMO for decortication. The overall ICU mortality rate was 24% (8 out of 34 patients). Ninety days after the patient was admitted, the overall mortality rate was 27% (9 out of 34 patients), and there was no significant difference between patients using ECMO and those not using it. Because heart failure during a pheochromocytoma crisis may be reversible, ECMO can maintain adequate organ perfusion until the recovery of cardiac function takes place.^65^ Novelis et al. reported the case of a patient with severe ischemic dilated cardiomyopathy and arterial infiltration in 2022. ECMO support was provided during the lobectomy and pulmonary angioplasty.^66^ In conclusion, by providing surgical resection, ECMO offers a glimmer of hope to patients who would otherwise be inoperable.

### 2.3 ECMO as a bridge to chemotherapy

In general, chemotherapy is not routinely recommended for patients with ECMO because of possible complications such as neutropenia or thrombocytopenia. However, according to ELSO regulations, chemotherapy for ECMO is not an absolute contraindication.^67^ Some patients who are sensitive to chemotherapy can achieve tumor reduction and have a better prognosis.

A 40‐year‐old woman was diagnosed with a ventricular large B‐cell lymphoma. She successfully transitioned to chemotherapy with cardiopulmonary support from VA‐ECMO to achieve a reduction in tumor load and survived for up to 1 year.^68^ In 2015, Allain et al. described a 65‐year‐old man newly diagnosed with primary cardiac lymphoma who was successfully treated with chemotherapy and supported by ECMO. Cardiogenic shock occurred during the course of treatment, and circulatory support was required from the ECMO. Emergency chemotherapy with cyclophosphamide, vincristine, and solu‐medrol was administered on day 8 of ECMO support, and complete recovery was achieved 6 months later.^69^ A young woman with choriocarcinoma recently presented with refractory circulatory failure following a pulmonary embolism. Chemotherapy was administered with extracorporeal membrane oxygenation support, and the prognosis was good. The authors noted that ECMO helps patients with pulmonary embolism to transition to surgical embolization and chemotherapy.^70^ A 21‐year‐old male with primary B‐cell lymphoma invading the trachea developed refractory dyspnea, requiring chemotherapy in conjunction with ECMO support. After completing four cycles of chemotherapy, the patient was asymptomatic. He was discharged with a Karnofsky score of 100.^71^ Positive efficacy of ECMO in chemotherapy‐sensitive oncology patients was observed in these cases. Therefore, ECMO should not be ruled out in the first instance as salvage therapy for cancer patients with stable disease who are eligible to receive full‐code ICU care immediately. Comprehensive and rigorous study designs, such as prospective studies, are required to determine the statistical significance and to help inform our conclusions in case reports.

## 3 CONCLUSION

In summary, as mentioned in the consensus, cancer is only a relative contraindication to ECMO and not an absolute contraindication. ECMO is applied as a support for cancer patients during surgeries, solid tumor patients with respiratory failure, or blood cancer patients in severe condition during chemotherapy. However, according to current studies, there is still a large proportion of oncology patients who do not benefit from ECMO. Thus, further thought should be given on how to assess oncology patients regarding their eligibility for ECMO. Such evaluations can be completed through multidisciplinary treatment efforts (e.g., in collaboration with oncologists and surgeons) and should make use of full codes and individualized treatment for patients admitted to the ICU requiring ECMO. This topic needs to be evaluated more comprehensively in rigorously designed and highly powered clinical studies. Future studies should enroll larger sample sizes than those that have been conducted to date and include research on efficacy, adverse events, and health economics.

## AUTHOR CONTRIBUTIONS

**Xiangnan Teng:** Data curation (equal); writing – original draft (equal); writing – review and editing (equal). **Jiali Wu:** Investigation (equal); writing – review and editing (equal). **Jing Liao:** Data curation (equal); writing – review and editing (equal). **Shanling Xu:** Methodology (equal); resources (equal); writing – review and editing (equal).

## FUNDING INFORMATION

Sichuan Province Key Clinical Specialty Construction Project.

## CONFLICT OF INTEREST STATEMENT

The authors declare that they have no competing interests.

## Data Availability

Upon reasonable request, the corresponding author will provide access to the datasets used in this study.
